# The Incidence and Predictors of Thromboembolic Events in Patients with Lung Cancer

**DOI:** 10.1155/2014/125706

**Published:** 2014-01-20

**Authors:** Bohdan Kadlec, Jana Skrickova, Zdenek Merta, Ladislav Dusek, Jiri Jarkovsky

**Affiliations:** ^1^Department of Respiratory Diseases and TB, Faculty of Medicine, Masaryk University and University Hospital, Brno, Czech Republic; ^2^Institute of Biostatistics and Analyses, Faculty of Medicine and Faculty of Science, Masaryk University, Brno, Czech Republic

## Abstract

Patients with lung cancer experience elevated risk of venous thromboembolism. Cancer patients with thrombosis have a shorter life expectancy and the occurrence of VTE worsens the quality of life and may delay, interrupt, or completely halt the cancer therapy. In a large cohort of lung cancer patients we monitored the incidence of venous thromboembolism and we identified groups of patients with the highest risk of venous thromboembolism suitable for antithrombotic prophylaxis, which could favourably affect their morbidity and mortality.

## 1. Background

The relationship between thrombosis and cancer is known for quite a long time. It was first broached in 1865 by the French internist Armand Trousseau who described migratory thrombophlebitis as a precursor to cancer (Trousseau's syndrome) [1]. Thromboembolic disorders have a wide spectrum of clinical manifestations. The most common and serious is venous thromboembolism, including pulmonary embolism and deep venous thrombosis. The risk factors for VTE [2] are defined by the still valid theory formulated by the German pathologist Rudolf Virchow more than 150 years ago. The incidence of thromboembolic disease in general population is relatively low, about 1–3 per 1,000 people annually [3]. Among the cancer patients, the occurrence of VTE is 4–7 times higher, depending on the type and the stage of cancer [4]. Of patients who develop thromboembolic disease, 20% are cancer patients. However, data on the occurrence of VTE in cancer patients is probably underestimated, judging by autopsy findings where pulmonary embolism or venous thrombosis is found in more than 50% of autopsies [5], while VTE detection in lifetime only amounts to 15% of cases, indicating substantial shortcomings in diagnosis. The presence of VTE also serves as a negative predictor of survival in cancer patients and increases the probability of their death 2–8 times. Developing thromboembolism complicates cancer treatment and is associated with a significantly poorer prognosis. As results of a large Danish cancer registry are revealed [6], the one-year survival of cancer patients with VTE is 12%, against 36% in cancer patients without VTE. However, the risk is not the same for all tumors; it is greatest in lymphomas and cancer of brain, lung, pancreas, stomach, ovary and kidney [7]. The risk of thrombosis is also associated with the histological type of tumor, for example, mucin-producing adenocarcinoma (stomach, pancreas, lung, and ovary) [8].

One of the most common manifestations of cancer is hypercoagulation (Table 1). The tendency to hypercoagulation associated with smaller wettability and antithrombotic activity of vascular endothelial cells facilitates the adhesion of circulating tumor cells, which is a prerequisite for further metastatic spread of the tumor [9]. Tumor cells can directly activate blood clotting, inducing procoagulant or suppressing anticoagulant properties of vascular endothelium, platelets, monocytes, and macrophages. Overproduction of procoagulation factors, particularly the tissue factor, also leads to increased production of thrombin [10]. Apart from direct involvement in coagulation and primary hemostasis, thrombogenesis can also be due to distortion by the tumor of vascular endothelium's function and integrity, leading to activation of coagulation or restriction of blood flow in the vessel oppressed by tumor, with subsequent accumulation of activated clotting factors and platelets. The emergence and growth of thrombus is also supported by such complications of cancer as organ damage, sepsis, changes in the internal environment, and therapeutic interventions directed against cancer (chemotherapy, hormonal therapy, antiangiogenic therapy, and central venous catheters) [11]. General factors such as age, immobilization, and surgery also contribute to the emergence of VTE in cancer patients [12–15]. Laboratory changes include the increase in fibrinogen concentration, reduced clotting times and, quite frequently, thrombocytosis. Thrombocytosis occurs in gastric or colon cancer patients and in lymphoma patients [16, 17]. The increase in platelets* per se* does not indicate hypercoagulation, but it can facilitate thrombosis if simultaneous activation of procoagulant factors occurs [18]. Platelets circulate in the inactive state, but upon stimulation by activators (thrombin, prostaglandins, ADP, epinephrine, and other substances produced by tumors) there may be an increase in their spontaneous aggregation and adhesiveness [19].

Deep vein thrombosis and pulmonary embolism are the most important manifestations of thromboembolic disease in cancer patients [20]. Thrombophlebitis migrans, or migratory superficial phlebitis, is less frequent than VTE, but often precedes—even by months—other clinical signs of malignancy. This is mostly observed in patients with pancreatic, breast, prostate, ovarian, and gastric cancer [21].

Disseminated intravascular coagulation associated with bleeding and thrombotic symptoms is a rare manifestation of malignancy, yet it is common in some types of leukemia. In solid tumors, it can be present in advanced stages of the disease [22].

## 2. Material and Methods

Patients diagnosed with primary lung cancer, verified either histologically or cytologically, were recruited at the University Hospital's Department of Pulmonary Diseases and Tuberculosis, Brno, from January 2006 to May 2010. We recorded basic demographic data, data on histology and clinical stage, information about frequent and significant comorbidities, and details of the initial cancer chemotherapy treatment, radiotherapy, targeted therapy, surgery, or best supportive care. Chemotherapy in most cases entailed a combination of platinum derivatives with gemcitabine, paclitaxel, vinorelbine, etoposide, or pemetrexed. Targeted drugs for first-line treatment of advanced nonsmall cell lung cancer were EGFR inhibitors, gefitinib and erlotinib, and angiogenesis inhibitor bevacizumab.

Hematological examinations were performed in the laboratories of the University Hospital's Department of Clinical Hematology in Brno. Laboratory parameters we followed were PT, aPTT, fibrinogen, D-dimers, total number of platelets, and total white blood cell count and hemoglobin. In patients with locoregionally advanced, unresectable or metastatic lung cancer, these parameters were evaluated prior to the first cycle of chemotherapy or targeted therapy. The diagnosis of thromboembolic events was done by standard diagnostic procedures; their risk was summarily determined as high [23].

Descriptive statistics was used for description of the data: absolute and relative frequency of each category for categorical variables; median and 5%–95% percentiles for continuous variables. The analysis of categorical variables was supplemented with an analysis of frequency tables.

The differences between levels of hemostatic factors according to various categories were tested by nonparametric Kruskal-Wallis test, which guarantees sufficiently robust results unaffected by outliers or nonnormal distribution of the original data. Relationships between categorical variables and relationships between categories resulting from dividing the patients according to different types of thromboembolic events, were tested by chi-square test of maximum likelihood. The differences in continuous variables between groups of patients were tested by the Mann-Whitney *U* test and the Kruskal-Wallis test.

In determining the boundaries of hemostatic factors according to occurrence of thromboembolic events, receiver operating characteristic (ROC) analysis was used; its results are described by the area under the ROC curve with 95% confidence interval, along with the *P* value of the test for differences from the expected area under the curve of 0.5.

To analyze the relationship between various patient characteristic traits and the occurrence of thromboembolic events, logistic regression was used; its results are described by the odds ratio, confidence interval, and statistical significance.

All testing was performed at the level of statistical significance of 0.05; calculations were made using the IBM SPSS Statistics 20 program (IBM Corporation, 2011).

## 3. Results

### 3.1. Description of the Patient Group (Table 2)

We included 950 patients—600 men and 350 women. The median age of the patients was 64 years. The most frequently occurring histologic subtype was squamous cell carcinoma (27.5%), followed by adenocarcinoma (23.8%), small cell carcinoma (18.4%), and nonsmall cell without further specification (NOS); the remaining 10% was less common types such as large cell, sarcomatoid, and mixed carcinoid cancer. In 34 patients (3.6%), the diagnosis was not confirmed by histology or cytology. Representation of different clinical stages was similar to that in the general population; that is, most patients had advanced stages III (33.3%) and IV (41.4%) while clinical stages I and II were relatively less common (9.7% and 9.2%, resp.). Most patients were smokers or ex-smokers (88.1%), only 11.9% nonsmokers. Among comorbidities, hypertension was most frequent (39.6%), followed by COPD (38.2%), diabetes mellitus (19.4%), cerebrovascular disease 9.6%, and heart failure (7.7%).

### 3.2. The Incidence of Thromboembolic Events

Among 950 patients, we found 91 thromboembolic events (9.6%), of which 80 (87.9%) were serious and 11 (12.1%) less serious. We recorded 34 cases of pulmonary embolism, 58 cases of deep venous thrombosis, and 13 cases with simultaneous occurrence of both diseases. Also found were 11 cases of superficial thrombophlebitis, which were rated as less serious. The most common sources of venous thrombosis were lower limbs (49, 74%) and superior vena cava (5, 11%); less frequent was thrombosis associated with implantable ports and venous catheters (3, 6%), while other sources (superior vena cava and subclavian vein) were rarely encountered.

### 3.3. Hemostatic Parameters

Plasma levels of hemostatic factors had no significant dependence on gender, histology, clinical stage, or smoking; only the age over 65 years appears to be statistically significant. As to comorbidities, statistically increased values of median platelet count were found in patients with COPD—304 × 109 (115.0–497.0) *P* = 0.031 and heart failure 340 × 109 (149.0–522.0) *P* = 0.020. In the group of patients with thromboembolic events, significantly higher levels of platelet count were found at the time of diagnosis of lung cancer—368.0 (191.0–540.0) *P* < 0.001 (Figure 2); also significantly higher was the median value of WBC 6.0 × 109 (2,3–10,7), *P* = 0.017. On the contrary, median values of PT, aPTT, fibrinogen, or D-dimers were not significantly increased.

### 3.4. Characteristic of Patients according to the Occurrence of Thromboembolic Events

When comparing groups of patients according to the occurrence of thromboembolic events, we found statistically significant proportion of adenocarcinomas in patients with thromboembolic events—37 (40.7%) *P* = 0.02, a smaller proportion of squamous cell carcinomas—19 (20.9%), and small cell carcinomas—14 (15.4%). Among treatment modalities, the highest incidence of thromboembolic events had patients undergoing radiotherapy—10 (11%), surgery—16 (17.6%) and chemotherapy 44 (48.4%). In the group of patients with thromboembolic events we detected a significantly higher proportion of patients with COPD—44 (48.4%) *P* = 0.038, diabetes mellitus—27 (29%) *P* = 0.013, cerebrovascular disease—20 (22%) *P* < 0.001, hypertension—54 (29.3%) *P* < 0.001, heart failure—32 (35.2%) *P* < 0.001, atrial fibrillation—13 (14.3%) *P* = 0.004, and obesity—14 (15.4%) *P* = 0.017.

### 3.5. Predictors of Thromboembolic Events

Statistical analysis of results (Table 3) reveals age as a major risk factor for thromboembolic events in patients with lung cancer. In patients aged 50–65 years, OR is 1.19 (0.158 to 2.44), in patients aged >65 years, OR is 1.50 (0.74 to 3.04), and in those aged over 80 years, OR is 2.11 (0.94 to 4.74). Other significant predictors of VTE are adenocarcinomas—OR 3.79 (1.13 to 12.73), and radiotherapy—OR 3.64 (1.69 to 7.84). Among comorbidities, heart failure was identified as most risky for development of thromboembolic events—OR 13.48 (7.80 to 23.28), followed by cerebrovascular disease—OR 3.17 (1.78 to 5.64), atrial fibrillation—OR 2.96 (1.50 to 5.83), and obesity—OR 2.40 (1.26 to 4.58). Among monitored laboratory parameters, platelet count above 330.5 × 109 was associated with OR for major thromboembolic events was 3.66 (2.25 to 5.96)—a statistically significant value (Table 4). OR for D-dimer levels >0.4 mg/L was found to be 1.65 (1.01 to 2.69) and OR for aPTT > 35.95 was 1.74 (1.088 to 2.79).

### 3.6. Evaluation of Patients with Thromboembolic Disease at the Time of Chemotherapy Initiation

**Figure 1:**
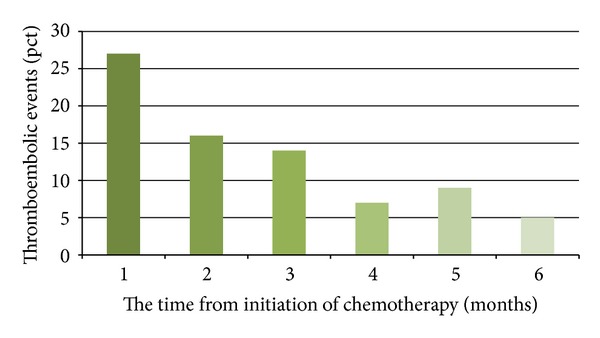
The occurrence of thromboembolic events after the start of chemotherapy.

**Figure 2:**
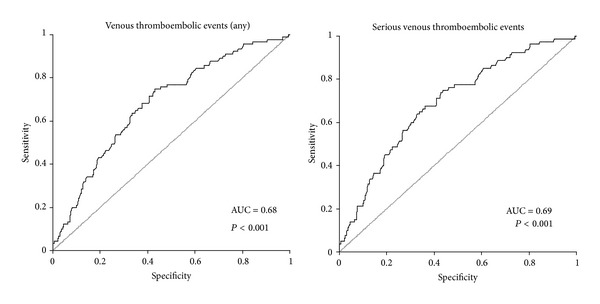
ROC analysis of the relationship between platelets and thromboembolic events.

The evaluated data shows that the majority of thromboembolic events in patients with lung cancer undergoing chemotherapy occur within 6 months of its initiation (Figure 1).

## 4. Discussion

In a large cohort of lung cancer patients, we observed a high incidence of VTE—8.4%, and we identified a number of risk factors: advanced stage of disease, adenocarcinoma, and comorbidities such as heart failure, atrial fibrillation, and cerebrovascular disease. The adjusted incidence rate is 21 cases per 1,000 people per year, which is at least seven times more than the incidence in the general population. This exceeds the incidence reported in previously published studies [7, 24], probably due to the higher number of complicated patients with advanced disease referred to the University Hospital. Improved methods of diagnosis can likewise contribute to the increased incidence.

Adenocarcinomas were associated with twofold increase of risk of VTE compared with squamous-cell carcinomas, a finding consistent with previously published data [25, 26]. In these studies, mucin-producing adenocarcinoma of the lung was associated with the highest risk of VTE; it is assumed that the presence of mucus may lead to increased secretion of procoagulant factors [27]. There is some evidence that mucin activates platelets and causes microthrombi to form in capillary circulation [26].

We found that in lung cancer patients advanced disease was a significant independent predictor of VTE, the incidence of thromboembolic events in patients with locoregional advanced or metastatic disease being nearly four times higher than in patients in early stages of the disease. This finding is supported by previously published data [5, 28].

An increased incidence of thromboembolic events was associated with the increased number of serious comorbidities (especially heart failure, atrial fibrillation, cerebrovascular diseases, and COPD). Similar trends were repeatedly described in noncancer populations [29, 30].

Elevated platelet count was shown to be another significant predictor of VTE in our group of patients associated with an increased risk of thromboembolic disease. Thrombocytosis was found in patients with advanced disease, more commonly in men, with no apparent correlation with the clinical stage of the disease [31].

No association was found between increased levels of D-dimer and other coagulation parameters monitored at the time of diagnosis in our patients. Pirk and colleagues found elevated plasma levels of D-dimer associated with an increased risk of death. The risk of death in patients with elevated D-dimer levels was independently increased in different subsets of patients, which is consistent with previous observations in small studies conducted in patients with cancer of breast [32, 33], colon [34, 35], and lung [36–38]. Relationship between elevated levels of D-dimer and poor prognosis of cancer patients was observed in hematologic malignancies and solid tumors, which is remarkable because high D-dimer was described as a predictor of VTE in earlier works. This shows that an increased activation of coagulation and fibrinolysis, as reflected in high D-dimer levels, is independently associated with poor prognosis and does not necessarily indicate an increased risk of VTE in patients with elevated D-dimer levels [39, 40].

Zecchina and colleagues [41], who investigated the activation of the hemostatic factors in 45 patients with advanced lung cancer in a prospective study, found several other mechanisms increasing the risk of thrombosis in patients with lung cancer. While there were no significant changes in such parameters as D-dimer, fibrinogen, and activated protein C, a significant increase in platelet count was found 21 days after the administration of chemotherapy, correlating with the occurrence of VTE. Another clinical study showed that patients with lung cancer had significantly higher levels of thrombin-antithrombin complexes III, D-dimer, and plasmin-alpha 1-antiplasmin complexes compared with nonmalignant populations, suggesting an increased activation of coagulation and fibrinolysis [42]. Elevated levels of procoagulant factors, such as lupus anticoagulant, anticardiolipin antibodies to factor VIII, and certain cytokines such as interleukin-6 and tumor necrosis factor, have also been identified as increased risk factors for VTE in patients with lung cancer [43].

Callander et al. [44] and Ornstein with coworkers [45] made an important finding that nonsmall cell lung carcinomas—squamous tumors and even adenocarcinomas—produced tissue factor (TF). Sato et al. [46] described a female patient with adenocarcinoma of the lung where tissue factor played a crucial role in the pathogenesis of recurrent VTE. In her case, plasma levels of TF were significantly elevated. Also, it was demonstrated that cancer cells contained TF factor, as evidenced by staining of tumor tissue using monoclonal antibodies—suggesting that cancer cells themselves could release TF into the circulation.

Khorana and colleagues [47] analyzed the data in a prospective, multicenter study of 3,003 cancer patients receiving at least one cycle of chemotherapy. VTE was found in 58 patients (1.93%), with the highest incidence of VTE in tumors of gastrointestinal tract (2.3%/month) and lung cancer (1.2%/month). An important finding was that the elevation of platelet levels before the start of chemotherapy was associated with a threefold increase in the incidence of VTE. In a multivariate analysis, the increased level of platelets prior to chemotherapy was statistically significantly associated with VTE. The majority of thromboembolic events occurred shortly after the initiation of chemotherapy; 18.1% of VTE occurred in the first month of chemotherapy, 47% within the first three months, and 72.5% within the first six months. These findings suggest that prevention of thrombosis should be considered in lung cancer patients undergoing chemotherapy who have elevated platelet levels.

In the treatment of lung cancer, recent years have seen a remarkable boom of targeted therapy, focused mainly on the EGFR inhibition with erlotinib and gefitinib, and neoangiogenesis inhibition with VEGF inhibitor bevacizumab. Data from clinical studies indicate that the use of angiogenesis inhibitors is associated with an increased incidence of VTE, but also with an increased incidence of bleeding. In our sample, the total number of patients treated with targeted (biological) therapy was too small to allow assessment of statistical significance. Combined modality treatment consisting of chemotherapy, radiotherapy, and the antiangiogenic agent thalidomide led in NSCLC patients to excessive toxicity as well as a higher incidence of thromboembolic events [48], as already seen in previous studies in other malignancies. Yoon and coworkers [49] reported that chemotherapy with bevacizumab, irinotecan, 5-fluorouracil, and leucovorin was associated with a high incidence of thromboembolism.

## 5. Conclusion

The observed incidence (8.4%) of serious thromboembolic events in patients with lung cancer is high, and it is even significantly higher in patients with nonsmall cell carcinomas, particularly in adenocarcinomas. Most thromboembolic events occurred in patients with advanced stage of the disease and in patients with comorbidities, especially heart failure. In patients with thromboembolic events we found significantly higher median platelet counts at the time of cancer diagnosis; with platelet count above 330,5 × 109, the odds ratio of severe VTE was 3.66 (2.25–5.96). Among comorbidities, heart failure was associated with the highest risk for development of VTE—OR 13.48 (7.80–23.28), followed by cerebrovascular disease—OR 3.17 (1.78–5.64), and atrial fibrillation—OR 2.96 (1.50–5.83). In patients with lung cancer, we did not find significantly higher medians of coagulation parameters (D-dimer, fibrinogen, thrombin time, and aPTT). In patients treated with chemotherapy, most VTE were observed shortly after treatment's inception (26.4% in the first month, with a gradually declining trend). The majority of thromboembolic events occurred within 6 months from the initiation of chemotherapy.

## Figures and Tables

**Table 1:** Prothrombotic mechanisms in tumors.

Proaggregation and procoagulant activity, increased platelet	
Acceleration of thrombin production, overproduction of tissue Factor, and other procoagulant factors	
Cytokine production by tumor cells, production of TNF-1, interleukin-11, and VEGF	
Direct interactions between tumor cells and the endothelium of blood vessels (increased adhesiveness)	
Factors related to cancer treatment—surgery, radiotherapy, chemotherapy, and antiangiogenic treatment	
Increased immobilization, central venous catheters	

**Table 2:** Patient population description.

Population description		
*N* = 950	*N* ^1^	Value^2^
Sex	950	
Male		600 (63.2%)
Female		350 (36.8%)
Age	950	64.0 (42.0–84.0)
<50		153 (16.1%)
50–65		356 (37.5%)
65–80		327 (34.4%)
>80		114 (12.0%)
Histology	950	
Adenocarcinoma		226 (23.8%)
Squamous cell carcinoma		261 (27.5%)
Small cell carcinoma		175 (18.4%)
Large cell carcinoma		61 (6.4%)
Small cell carcinoma not otherwise specified		113 (11.9%)
Other		80 (8.4%)
Not determined		34 (3.6%)
Clinical stage	823	
I		80 (9.7%)
II		76 (9.2%)
III		274 (33.3%)
IV		393 (41.4%)
Initial treatment	950	
Chemotherapy		605 (63.7%)
Radiotherapy		45 (4.7%)
Surgery		149 (15.7%)
Biological therapy		25 (2.6%)
Symptomatic treatment		126 (13.3%)
Smoking	907	799 (88.1%)
COPD	950	363 (38.2%)
Diabetes mellitus	950	184 (19.4%)
Cerebrovascular disease	950	91 (9.6%)
Hypertension	950	376 (39.6%)
Heart failure	950	73 (7.7%)
Atrial fibrillation	950	61 (6.4%)
Obesity	950	78 (8.2%)
Platelets at the time of diagnosis	950	286.5 (107.0–488.0)
WBC	950	7.0 (1.9–12.5)
Hemoglobin	950	122.0 (93.0–154.0)
PT	926	1.0 (0.7–1.7)
aPTT	925	34.5 (24.6–44.4)
Fibrinogen	874	3.1 (1.8–5.7)
D-dimers	811	0.4 (0.2–0.6)
Thromboembolic event 6 months after treatment initiation	950	91 (9.6%)
Severity of thromboembolic event	91	
Nonserious		11 (12.1%)
Serious		80 (87.9%)

^1^Available *N*.

^2^Categorical variables are described by absolute and relative frequencies; continuous variables are described by median and 5%–95% percentiles.

**Table 3:** Relationship between potential predictors and occurrence of thromboembolic events.

*N* = 950	Any VTE	OR (95% CI)^1^	Serious VTE	OR (95% CI)^1^
Sex (*N* = 950)				
Male	50 (8.3%)	Reference	43 (7.2%)	Reference
Female	41 (11.7%)	1.46 (0.94–2.26)	37 (10.6%)	1.53 (0.97–2.43)

Age		1.02 (1.00–1.04)*		1.02 (1.00–1.04)*
Age categorized (*N* = 950)				
<50	11 (7.2%)	Reference	10 (6.5%)	Reference
50–65	30 (8.4%)	1.19 (0.58–2.44)	24 (6.7%)	1.03 (0.48–2.22)
65–80	34 (10.4%)	1.50 (0.74–3.04)	31 (9.5%)	1.50 (0.71–3.14)
>80	16 (14.0%)	2.11 (0.94–4.74)	15 (13.2%)	2.17 (0.94–5.02)

Histology (*N* = 950)				
Adenocarcinoma	37 (16.4%)	3.79 (1.13–12.73)*	32 (14.2%)	3.19 (0.94–10.79)
Squamous cell carcinoma	19 (7.3%)	1.52 (0.43–5.30)	18 (6.9%)	1.43 (0.41–5.03)
Small cell carcinoma	14 (8.0%)	1.68 (0.47–6.06)	11 (6.3%)	1.30 (0.35–4.81)
Large cell carcinoma	3 (4.9%)	Reference	3 (4.9%)	Reference
NSCLC NOS	9 (8.0%)	1.67 (0.44–6.43)	8 (7.1%)	1.47 (0.38–5.77)
Other	6 (7.5%)	1.57 (0.38–6.54)	5 (6.3%)	1.29 (0.30–5.62)
Not determined	3 (8.8%)	1.87 (0.36–9.83)	3 (8.8%)	1.87 (0.36–9.83)

Clinical stage (*N* = 823)				
I	9 (11.3%)	Reference	7 (8.8%)	Reference
II	11 (14.5%)	1.34 (0.52–3.43)	10 (13.2%)	1.58 (0.57–4.39)
III	29 (10.6%)	0.93 (0.42–2.06)	25 (9.1%)	1.05 (0.44–2.52)
IV	36 (9.2%)	0.80 (0.37–1.72)	32 (8.1%)	0.92 (0.39–2.18)

Initial treatment (*N* = 950)				
Chemotherapy	44 (7.3%)	Reference	39 (6.4%)	Reference
Radiotherapy	10 (22.2%)	3.64 (1.69–7.84)*	9 (20.0%)	3.63 (1.63–8.07)*
Surgery	16 (10.7%)	1.53 (0.84–2.80)	13 (8.7%)	1.39 (0.72–2.67)
Targeted therapy	3 (12.0%)	1.74 (0.50–6.04)	2 (8.0%)	1.26 (0.29–5.55)
Symptomatic treatment	18 (9.6%)	2.13 (1.18–3.82)*	17 (13.5%)	2.26 (1.24–4.15)*

Smoking (*N* = 907)	81 (10.1%)	1.11 (0.56–2.20)	71 (8.9%)	1.07 (0.52–2.21)
COPD (*N* = 950)	44 (12.1%)	1.59 (1.03–2.45)*	39 (10.7%)	1.60 (1.01–2.54)*
Diabetes mellitus (*N* = 950)	27 (14.7%)	1.89 (1.17–3.05)*	25 (13.6%)	2.03 (1.23–3.36)*
Cerebrovascular disease (*N* = 950)	20 (22.0%)	3.13 (1.80–5.43)*	18 (19.8%)	3.17 (1.78–5.64)*
Hypertension (*N* = 950)	54 (14.4%)	2.43 (1.57–3.78)*	49 (13.0%)	2.63 (1.64–4.20)*
Hearth failure (*N* = 950)	32 (43.8%)	10.82 (6.35–18.43)*	32 (43.8%)	13.48 (7.80–23.28)*
Atrial fibrillation (*N* = 950)	13 (21.3%)	2.82 (1.46–5.42)*	12 (19.7%)	2.96 (1.50–5.83)*
Obesity (*N* = 950)	14 (17.9%)	2.26 (1.21–4.21)*	13 (16.7%)	2.40 (1.26–4.58)*

Platelets at the time of diagnosis (*N* = 950)		1.01 (1.00–1.01)*		1.01 (1.00–1.01)*
PLT > 299.5	68 (15.5%)	3.89 (2.38–6.36)*	—	—
PLT > 330.5	—	—	54 (14.6%)	3.66 (2.25–5.96)*
WBC (*N* = 950)		0.92 (0.86–0.99)*		0.93 (0.86–0.99)*
WBC < 6.36	51 (12.9%)	1.92 (1.24–2.97)*	43 (10.9%)	1.72 (1.09–2.72)*
Hemoglobin (*N* = 950)		0.99 (0.98–1.00)		0.99 (0.98–1.00)
Hemoglobin < 123.5	53 (10.3%)	1.20 (0.78–1.87)	46 (8.9%)	1.16 (0.73–1.85)
PT (*N* = 926)		0.96 (0.49–1.86)		0.89 (0.43–1.83)
PT < 1.05	48 (9.8%)	1.10 (0.70–1.71)	43 (8.7%)	1.16 (0.73–1.87)
aPTT (*N* = 925)		1.02 (0.99–1.06)		1.03 (0.99–1.07)
aPTT > 35.95	45 (11.7%)	1.59 (1.02–2.47)*	41 (10.7%)	1.74 (1.08–2.78)*
Fibrinogen (*N* = 874)		1.08 (0.90–1.28)		1.09 (0.91–1.31)
Fibrinogen > 3.15	47 (11.2%)	1.52 (0.96–2.41)	—	—
Fibrinogen > 3.35	—	—	37 (10.0%)	1.48 (0.91–2.40)
D dimers (*N* = 811)		0.97 (0.82–1.15)		0.97 (0.81–1.17)
D dimers > 0.40	32 (11.9%)	1.65 (1.01–2.69)*	—	—
D dimers > 0.38	—	—	31 (9.2%)	1.41 (0.84–2.36)

^1^Logistical regression.

*Statistically significant value OR (*P* < 0.05).

**Table 4:** Hemostatic factors “cut-offs” for venous thromboembolic events.

	Any VTE					Serious VTE				
	AUC (95% CI)^1^	*P*	Sensitivity	Specificity	Cutoff	AUC (95% CI)^1^	*P*	Sensitivity	Specificity	Cut Off
Platelets	0.68 (0.63–0.74)	<0.001	0.747	0.568	299.5	0.69 (0.63–0.75)	<0.001	0.675	0.638	330.5
WBC	0.58 (0.52–0.63)	0.017	0.560	0.602	6.36	0.57 (0.51–0.63)	0.042	0.538	0.598	6.36
Hemoglobin	0.54 (0.48–0.61)	0.180	0.582	0.463	123.5	0.54 (0.47–0.61)	0.261	0.575	0.462	123.5
PT	0.51 (0.44–0.57)	0.835	0.552	0.487	1.05	0.51 (0.44–0.58)	0.751	0.566	0.488	1.05
aPTT	0.54 (0.48–0.61)	0.194	0.517	0.597	35.95	0.55 (0.48–0.62)	0.148	0.539	0.597	35.95
Fibrinogen	0.53 (0.46–0.60)	0.435	0.573	0.532	3.15	0.53 (0.45–0.60)	0.480	0.514	0.584	3.35
D dimers	0.56 (0.48–0.63)	0.117	0.438	0.679	0.40	0.54 (0.46–0.63)	0.249	0.492	0.592	0.38

^1^ROC analysis.

## Abbreviations

aPTT: Activated partial thromboplastin time
ADP: Adenosine diphosphate
AT: Antithrombin
CI: Confidence interval
CNS: Central nervous system
EGFR: Receptor for epidermal growth factor
DVT: Deep vein thrombosis
OR: Odds ratio
PC: Protein C
PE: Pulmonary embolism
PT: Prothrombin time
ROC: (Receiver operating characteristic) curve is based on a pivot table right, respectively, wrong classification of positive or negative sample
SCLC: Small cell lung cancer
NSCLC: Nonsmall cell lung cancer
NOS: Not otherwise specified
RF: Risk factor
RR: Relative risk
VTE: Venous thromboembolism.
